# Patient perspectives on depot buprenorphine treatment for opioid addiction – a qualitative interview study

**DOI:** 10.1186/s13011-022-00474-2

**Published:** 2022-05-25

**Authors:** Björn Johnson, Olivia Liahaugen Flensburg, Andrea Johansson Capusan

**Affiliations:** 1 Department of Social Work, Malmö University, Malmö, Sweden; 2 Department of Psychiatry in Linköping and Department of Biomedical and Clinical Sciences, Linköping University, Linköping, Sweden; 3 Center for Social and Affective Neuroscience, Department of Biomedical and Clinical Sciences, Linköping University, Linköping, Sweden

**Keywords:** Opioid addiction, Treatment, Depot injections, Pharmaceutical atmosphere, Qualitative interviews

## Abstract

**Background:**

Recently developed buprenorphine depot injections have the potential to reduce risk for diversion and misuse, and to increase adherence with fewer visits for supervised intake. However, it is unclear how patients perceive this new form of medication. The purpose of this study was to explore patients’ experiences of depot injections and their reasons for continuing, discontinuing, or declining depot injection treatment.

**Methods:**

We conducted semi-structured qualitative interviews with 32 people, 14 of whom had ongoing depot injection treatment, 11 who had discontinued depot-injections and switched to other medication and seven who had declined treatment with depot formulations. Interviews were transcribed, coded, and analysed using NVivo, based on this overall stratification into three participant groups.

**Results:**

The main categories relate to the effects and side effects of the depot formulation, social and practical factors, psychological benefits and disadvantages, and interactions with treatment staff. Social and practical factors were of importance for choosing depot formulations, such as increased freedom and their making it easier to combine treatment with work and family life, as well as psychological advantages including “feeling normal”. Initial withdrawal symptoms that resolved themselves after a number of injections were reported by most participants. Reliable information and patient-staff relationships characterized by trust helped patients to cope with these initial problems. Those who discontinued treatment often did so near the beginning of the treatment, reporting withdrawal symptoms and insufficient effects as the main reasons. Coercion and insufficient information contributed to a negative pharmaceutical atmosphere at one of the clinics, which may have adversely influenced perceptions of depot formulations and decreased willingness to accept and continue treatment.

**Conclusions:**

Buprenorphine depot injections may have social, practical, and psychological benefits compared to other formulations. However, depot injections are not perceived as an attractive option by all patients. Trust, consistent and adequate information, and awareness of the implications of the pharmaceutical atmosphere should be considered when introducing new medications.

**Supplementary Information:**

The online version contains supplementary material available at 10.1186/s13011-022-00474-2.

## Background

Opioid addiction is a disorder characterized by high morbidity and mortality [1–4]. Opioid agonist treatment (OAT) with methadone or buprenorphine is highly effective in opioid addiction [5–7], reducing all-cause mortality [8], and is recommended for those with opioid addiction in both international [9, 10] and national [11, 12] guidelines.

However, a lack of staff and other resources still limit access to treatment in Sweden [13], with some patients waiting several months, or up to years, after a decision on treatment, before they are actually offered medication [12]. The situation has been exacerbated by strict governmental regulations regarding inclusion and exclusion criteria, patient attendance, medication collection routines, and urine testing [14]. Daily supervised intake of medication over several months has traditionally been a common strategy to reduce diversion. However, while failing to prevent diversion, which is quite common even in supervised treatment programs [15], high levels of control may lead to mistrust between patients and staff, and may prevent patients from seeking treatment or adhering to treatment programs [16].

Buprenorphine depot injections, recently developed and approved for once weekly [17] or monthly administration [17, 18], open up for the possibility of fewer visits to clinics, and thus the possibility of providing more patients with access to treatment. Depot injections have shown an efficacy equal to that of oral buprenorphine [17] and better than placebo [18], with no risk for diversion or misuse, and consequently less need for control. However, we know little about how depot injections will affect patients’ everyday lives, how they fit into the wider frame of rehabilitation and recovery, or how they influence patients’ relationships with treatment staff.

Qualitative research brings users’ perspectives into the field of addiction treatment [19]. While several studies conducted prior to the introduction of depot buprenorphine explored patients’ attitudes, information needs, and willingness to receive depot medications [20–23], only one previous study has examined the perspectives of patients on receiving depot injections in an OAT setting [24]. This qualitative study is based on interviews with 30 Australian patients about their experiences of buprenorphine depot injections. The results indicated that depot injections benefited many participants. Fewer visits to clinics and pharmacies meant reduced stigma, more time to engage in other activities (work, leisure, and travel) and cost savings from reduced pharmacy fees. However, some participants also mentioned negative aspects, including disrupted engagement with the social and practical support offered at clinics and pharmacies, reduced ability to control dosing, and that they could no longer make money by selling takeaway doses.

An open label RCT conducted in Australia [25] indicated higher treatment satisfaction, measured with the Treatment Satisfaction Questionnaire, in 60 patients receiving depot injections, compared with 59 treated with sublingual buprenorphine naloxone. Although injections site reactions were more frequent of those treated with depot injections (65%) compared to those treated with sublingual formulations (20%), no patients discontinued trial medication due to adverse events. However, clinical experience indicates that a number of patients who start depot injections discontinue treatment. No previous qualitative study has explored their perceptions on depot medication.

According to a qualitative interview study in the UK prior to depot injections becoming available [22], both patients in OAT and currently untreated users with daily heroin intake were interested in depot injection treatment. Several factors that potentially influence the willingness to receive long-acting formulations were identified, such as reduced contact with services, impacts on illicit drug use and recovery, perceived effectiveness, duration and dose of depot injections, and potential side-effects associated with depot injections.

Focus groups also conducted prior to depot injections becoming available, comprising participants with OAT experience, identified potential benefits such as increased stability and freedom, but also raised potential concerns such as a loss of interactions with treatment providers and of necessary support [20]. The study viewed medication as a factor that interacts in a broader context with other relevant social, medical, and psychological factors, such as the breaking of rituals and habits, feeling normal, and getting on with normal life versus losing social interactions with treatment staff [20]. The same UK research group explored patients’ information needs regarding depot injections. Based on qualitative interviews with patients both in and outside treatment, researchers recommend accessible biomedical and lay information on depot buprenorphine to enable patients to consider their options and participate in treatment decision making [21].

Not all patients expressed however a willingness to receive depot injections, prior to them becoming available. A cross-sectional survey conducted in Australia [23] using computer-based structured interviews found that around two-thirds of 402 opioid users, 255 of whom were currently in OAT, considered long-acting buprenorphine a potentially good treatment option, while another survey on inpatients treated for opioid withdrawal found that only around one quarter expressed an interest in trying a long-acting buprenorphine formulation [26]. No prior study has interviewed patients who had been offered depot injections and declined.

Previous research indicates that the local contexts, or “pharmaceutical atmosphere” as described by medical anthropologist Aleksandra Bartoszko, may influence perceptions of a certain medication [27]. This pharmaceutical atmosphere, emerging in the tension between clinical practice, the regulatory framework, scientific narratives, and the patients’ emotions, history, and experience, may in turn influence the perception of efficacy and side effects. Analyzing the introduction of buprenorphine-naloxone combination tablets in Norway, when patients were forced to transfer from buprenorphine to combination treatment, Bartoszko described how an inadequate introduction “polluted the therapeutic atmosphere” of OAT [27].

The purpose of this study is to explore how patients at OAT clinics in a European setting, in Sweden perceive treatment with buprenorphine depot injections. To this end we conducted semi-structured in-depth interviews focused on how depot injections impact the patients’ everyday lives, contacts with caregivers, and attitudes towards OAT treatment and services. In contrast to earlier study [24], including only participants currently treated with depot injections, we interviewed a wider group of patients in order to explore their reasons for continuing, discontinuing, or declining depot injections in favor of other treatment alternatives. Interviews include participants from several centers, working independently, enabling us to detect a potential impact of the local “pharmaceutical atmosphere”.

## Materials and methods

The study is part of a follow up of the clinical introduction of depot buprenorphine in Sweden initiated at the Addiction Clinic at Linköping University Hospital. Subjects were recruited from seven different opioid addiction treatment facilities throughout Sweden (Höör, Jönköping, Linköping, Lund, Umeå, Ängelholm, Östersund), covering urban and rural areas in both the more densely populated southern Sweden and the sparsely populated areas in the north, where some of the patients travel several hours every day to collect their supervised medication doses.

The first buprenorphine depot injection was approved in Sweden in February 2019 (Buvidal weekly and monthly injections). In 2020, Sublocade (in Sweden known as Subutex Depot Injection) was also approved, but is not yet available on the market, although some clinics have a special permit from the Swedish Medical Products Agency to administer Sublocade. Thus, depot injections in this study refer primarily to weekly or monthly injections of Buvidal, except for one participant, who was receiving Sublocade depot injections.

### Sampling and participants

Qualitative, semi-structured interviews were conducted between May 2020 and March 2021 at seven OAT programs in as many Swedish cities and towns. A total of 32 participants were interviewed. The inclusion criteria were that the participants should be enrolled in OAT and should also have been offered or started treatment with buprenorphine depot formulation, willing to participate in the study, and able to speak and read Swedish sufficiently to provide written informed consent and participate in the interview conducted in Swedish.

Recruitment was based on purposive, stratified sampling [28]. We aimed to include participants from three different groups: 1) patients undergoing treatment with depot formulation (*n* = 14), 2) patients who had discontinued treatment with depot formulation and switched to another drug (*n* = 11), and 3) patients who had declined treatment with depot formulation (*n* = 7). Recruitment continued until data saturation was reached in the three groups, i.e. when no new relevant information emerged in additional interviews [29]. The participants were given the opportunity to voluntarily start treatment with depot formulation at the clinics included in the study, with the exception of one clinic, where the participants felt that they had been coerced into switching to depot formulation. Several of these participants stated that they would have declined if they had been given the choice. These participants are formally included in groups 1 and 2 above, but parts of the information they provided have been sorted under group 3 in the analysis.

### Interview procedure

Semi-structured interviews were conducted using an interview guide (Supplementary material 1) developed based on themes identified in qualitative studies undertaken prior to the introduction of depot injections in Europe (20), and on one of the researchers’ clinical experience (AJC). Themes covered: (a) background and history of substance use, (b) previous treatment experiences, (c) experiences with and views about OAT, (d) relationships with treatment staff, (e) views on control and support in the ongoing treatment, (f) thoughts about the choice of drug formulation, (g) perceptions of the information provided by staff about the depot formulation, and (h) thoughts on the future. Using semi-structured interviews allows the interviewer to clarify questions relating to the selected themes and helps participants provide more in-depth information.

All interviews were conducted by OLF, a research assistant with extensive experience of qualitative interviews in the field of opioid addiction.

Two weeks before the data collection, posters were posted at the clinics, and clinical staff briefed about the study. Clinical staff informed patients who were part of the target group about the opportunity to participate. Interested patients provided their phone numbers, which were then passed on to OLF, who contacted the patients to schedule the interviews.

The original intention was to conduct interviews on site at the clinics. However, the Covid-19 pandemic prevented both travel and visitors at healthcare facilities during parts of the data collection period. Interviews at certain clinics were therefore conducted by phone. A total of 19 face-to-face interviews and 13 phone interviews were conducted. Face-to-face interviews were conducted at the clinics, adjacent healthcare premises, and in one case in a park.

Prior to the interviews, participants received oral and written information about the study. They were told that the interviews would be confidential, that all data would be anonymized before publication, that participation would not affect the participants’ treatment in any way, and that they could discontinue the interview at any time. Before being interviewed, participants provided written informed consent, either to the interviewer, in the case of face-to-face interviews, or to research staff at the clinic in the case of telephone interviews. After completing the interviews, the participants received shopping vouchers for SEK 200 (approx. € 20).

The interviews lasted on average 41 minutes. Remote interviews were slightly longer than face-to-face interviews (46 versus 39 minutes on average). All interviews were recorded on a digital voice recorder and then transcribed verbatim by OLF. No differences in data quality were noted between the face-to-face and remote interviews, which is consistent with research on interview methods [30].

### Analysis

The transcribed interviews were first read through and then entered into NVivo (Release 1.5, QSR International 2021) for further analysis. A basic coding was performed inductively by BJ. This generated over 300 individual codes, but many of these were overlapping and/or occurred only once or twice. Case notes were kept continuously during the basic coding (and later during the coding process), in which interesting observations, patterns in the data, and possible interpretations were noted. In the next step, all the codes and the corresponding text sections were reviewed carefully, with some codes being removed and others being merged. The coding was then discussed with OLF. The coding process then moved on to create more general categories and subcategories, into which relevant codes were aggregated. The material was then analyzed based on the overall stratification into three groups of participants. In the last step of the analysis, the categories and codes were reviewed one last time, with illustrative quotes being selected from the corresponding texts.

## Results

### The participants

A total of 32 participants were interviewed, the majority (69%) male, with a mean age of 36.6 years (range 21–67) (Supplementary material 2). This sex and age distribution is consistent with a national survey of OAT in Sweden [31]. All participants had a diagnosed opioid dependence, with on average 14.5 years (range 6 to 29 years) of illicit substance use. The main substance of use prior to entering OAT had been heroin or heroin in combination with other opioids. Two individuals reported other main illicit substances (benzodiazepines and cannabis, respectively), but also reported daily use of opioids.

Twenty participants, had extensive experience of other, abstinence-oriented treatment interventions before starting OAT, including both in- and outpatient treatment and residential care, and in many cases also compulsory care. Many described a shorter or longer period of abstinence following these treatment options but had subsequently relapsed into illicit substance use. Ten participants had more limited abstinence-oriented treatment experience, and two participants had started OAT with no other treatment experience.

Many participants had relatively limited experience of OAT, on average 3 years (range: 2 months to 20 years), with 14 participants reporting less than 2 years in treatment. Of those who had ongoing depot treatment, nine were on monthly injections and five on weekly injections. Among those who had declined or discontinued depot formulations, the majority were receiving treatment with sublingual buprenorphine (12 participants) or buprenorphine-naloxone (5 participants).

At the time of the interview, 12 individuals stated that they were stably abstinent (> 12 months) from illicit substance use, 11 had been abstinent for a shorter period, and eight had ongoing use. For one person, we had no information on current use. In this respect, the participants differ significantly from an average group of patients in OAT in Sweden, where some level of illicit substance use is more the rule than the exception. The reason for this difference was that the depot formulations had mainly been offered, at certain clinics, to stable patients with no ongoing illicit substance use.

With a few exceptions, participants were positive to OAT. A substantial majority perceived staff at their clinic, as friendly, helpful and supportive, and felt that staff treated them well and wanted to do a good job. Most participants expressed confidence in the staff, and several spoke about how important it was to feel that they could be honest and talk about relapse and other problems, without risking punishment or other sanctions. There was however a minority who were dissatisfied with staff conduct and who felt that they were treated with distrust and unmotivated suspicion. This dissatisfaction was found among patients at several the clinics included in the study but was particularly common at one clinic. Several of those dissatisfied with clinic staff expressed a fear of punishment, for example by having their dose reduced, their medication withdrawn, or being discharged from treatment.

The main categories that emerged regarding participants’ perceptions of the depot formulation relate to their effects and side effects, social and practical factors, psychological benefits, and disadvantages. We start by describing these categories and then present the considerations constituting the reasons for participants’ decisions to decline, start, continue, or discontinue depot treatment.

### The effects and side effects of the depot-formulation

The effects, initial and more long-term side effects associated with the depot formulation were discussed at all interviews with participants who had accepted this type of treatment. Almost all reported experiencing initial withdrawal symptoms, which is to say that the weekly dose had initially not been sufficient for the entire week. Many had experienced this as difficult, but the majority had been informed by staff that they might experience withdrawal symptoms and had also been offered a shorter interval between doses during the adjustment period. A period of five or 6 days between doses was not unusual during the first weeks. Some said that they had also experienced other transient side effects – fatigue, nausea, restlessness, and difficulties sleeping – particularly in the first days following the depot injections.

Most of the participants who had continued with depot formulation described that withdrawal symptoms decreased after a few weeks and that the dose was now sufficient for the entire period that it was intended to last. *“Yes, I think we started on 24 [milligrams]. And then, it probably took about a month before I felt it was giving enough of an effect. [ …*] *I didn’t know what to expect of course, so I was happy with what I was getting so to speak (brief laughter). But then, yes, I mean I got used to the idea that it would take a while before the levels caught up, and then they did [catch up].”* Almost all participants stated that they were satisfied with the depot formulation, and several said that the effect was more even and stable than that from sublingual tablets or films. *“I feel fairly stable the whole time. There’s none of this up and down, [of the medication effect] but rather it’s even.”*

Experiences were different among the participants who had discontinued depot treatment. Several had done so after a relatively brief period on depot injections, due to withdrawal symptoms and/or drug cravings. But several also spoke of lasting withdrawal symptoms. These participants had attempted to continue with depot treatment for weeks or even months before switching to a sublingual buprenorphine formulation. *“Yes, those first days were totally ok, you know. But then I started to think; this was before it got to Monday again, you know. Then the whole weekend was crap, so to speak, and I lay there totally screwed, you know. Then of course I got one [injection], usually I got one every five days, on a rolling schedule. That worked fairly well. Mm. Then we did it with the monthly injections and all that, but that went straight to hell, it didn’t work.”* For some, the drug cravings had become so powerful that they had relapsed into the use of benzodiazepines or illicit opioids.

Most of the participants who had discontinued the treatment also described other negative experiences of the depot formulation. Several had developed lumps at the injection sites, on their stomachs or their arms. These had been unpleasant, particularly if they had taken a long time to disappear. *“People could think that you were ill, you know; you might think you had bubonic plague or something. There were big lumps all over my stomach.”* For some participants, such lumps had contributed to their choosing to discontinue the treatment. Many also described experiencing pain in connection with the injections. Lumps and pain of this kind were also mentioned by several of those currently on depot treatment, but these participants described them as something they were able to cope with, since the benefits of the treatment outweighed the drawbacks. Some participants who had declined the depot formulation also mentioned lumps as an argument for not testing the treatment – either because they had heard about them, or had seen lumps on other patients.

### Social and practical factors

Study participants often described social and practical factors as reasons for choosing to try the depot formulation. Many spoke of practical benefits and of the freedom that not having to visit the clinic as often to collect medication could give them. Several said that they were in employment, and that for this reason it felt good to avoid the stress of having to find time to visit the clinic. Having more time for family and leisure activities was also described as a benefit. *“It’s made a massive difference, I would say, for the better. Because I, I know that I can book in more or less as many work sessions as I want to now, except for like one day a week. I have much more time with my partner, I have a lot of time to other things, and it doesn’t feel like, it hardly feels like I’m on OAT anymore.”* Others mentioned long travel times to get to the clinic, and that they saved a lot of time by not having to make the journey as often. *“It was just this, that I don’t have to come here all the time. I don’t have to travel to and from [name of the town] like, and like the convenience.”* Some patients made a point of raising the fact that the depot formulation meant that they would be able to travel long distances, including trips abroad, to meet family and friends, which had previously been difficult, given that it takes many months in treatment, without relapse, before patients are entrusted with take-home doses to last for the whole journey.

Interestingly, there were also some participants who didn’t consider that depot formulation had led to any practical benefits. These were mostly individuals who had previously collected their medication from a pharmacy and who only visited the clinic a couple of times each month.

Many also spoke of social factors related to clinic visits. Opinions varied, however. Among those who agreed to take the depot formulation, many saw *“not having to get here [to the clinic] as often”* as one of the benefits. These participants were tired of the routine visits, which they felt did not give them anything. Others mentioned that it was good not to have to meet patients who were intoxicated, or who offered to sell them drugs – an issue we will return to in the section on the psychological benefits of the depot formulation.

However, there were also many participants who described their clinic visits as something positive or necessary in their lives. Some said that these visits constituted a fixed routine, which gave structure to their everyday lives. *“I’ve always thought it’s felt really good to be able to come here and talk to the staff. Not only because I feel that I can; it makes it more difficult to sneak around taking drugs, but also because it, it feels good to have a routine where you meet someone every morning when you’re going to like give up drugs.”* Others described their visits to the clinic as a valued social activity that contributed to decreasing their loneliness and isolation. *“It sounds a bit sick, but I mean it’s almost the only social life that I have, when I collect my medication, I mean. Since I’ve been at it for so many years, all my old friends and so on, they’re not around anymore.”* This type of story was found among all categories of participants, and some of those who had agreed to take the depot formulation mentioned that they had, at least to begin with, come to an agreement with the staff to continue visiting the clinic several times a week.

Other benefits of the depot formulation were not having to handle medication or have it at home. Some said that it felt good not to have to worry about their children getting hold of their medication. The fact that it was not possible to sell the depot formulation or share it with others was also discussed in the interviews. Several participants described this as something good and as giving peace of mind, since it meant that they did not have to deal with badgering and social pressure to show solidarity with those who wanted to buy their medication. *“Several have written and phoned and asked me like, ‘Yeah, I heard that you were on the sub-program now, can you like get me, or sell me a little?’ And then I: ‘No, I mean I get injections, just like when you inject a drug or something, so I get it in the arm and I can’t, for then it is where it is, so I can’t … ’. And that gets passed on to others, and then they don’t even ask.”*

### Psychological benefits – a shift in self-perception

One recurrent theme among the participants who had chosen to take the depot formulation was the psychological benefits that this type of treatment could provide. For these participants, the depot formulation could contribute to a shift in their self-perceptions, away from an identity as a drug user and a patient. The participants described it as being able to feel more “free” and “normal”, *“a bit more like ordinary people”. “I think that I don’t feel in the same way, like I’m trapped or something like that; I think I feel free in a different way.”* The depot formulation led to them being able to “move on”, both in their treatment and in life. This had been an important motivation for several of the participants who agreed to the depot formulation. *“I wanted to move on with, move on with the treatment, or how can I put it, so that I’m not one of those people who just sits and takes their methadone every morning.”*

Many participants described relief at not having to think about their medication every day. *“When I wake up, I don’t have to think about having to take some medication sort of thing. And that feels so bloody good. So, I can just get up and jump in the shower or, and do whatever, without needing to think about that I have to stand and freeze half to death and that.”* Several participants described that needing to take an opioid medication every day could serve as a constant reminder of their time as substance users. They were relieved at not having to experience this anymore. Having a depot injection each week or each month was *“not the same drug behavior [as] getting up every morning and taking something just to get through the day. That’s what I did before, of course when I was taking oxycontin; then I took oxycontin to cope with going to work. And I’m quite happy that I don’t have that pattern anymore; it’s better just to have it in your body.”*

Several participants said that the depot formulation reduced the drug stigma associated with having treatment for opioid addiction. Those who spoke of this were not comfortable with being open about receiving OAT or felt that their treatment had nothing to do with anyone else. *“I mean then you don’t have to hide it as much, you know if you meet someone or something like that; it’s like, with new people and so on, I never tell anyone that I’m in OAT.”* With the depot medication, there was no need to conceal medications and repeated visits to the clinic, and you did not have to deal with difficult questions: *“Nobody at all needs to know that I’m taking buprenorphine.”*

Another psychological benefit that many mentioned was that they were less often exposed to the risk environment that may be associated with OAT clinics. At the clinic, there is a risk of meeting persons in active drug use, and it is often possible to purchase illicit drugs or medications. This may constitute a severe temptation for individuals who do not feel particularly stable. *“Then it’s been a bit tough every now and again because a lot of my old contacts come here, so to speak, specifically those with abuse. And an awful lot of those who are here are active abusers. And that’s quite difficult. Just the fact that it’s so close, and I become a bit scared for myself, that I’ll get back into it.”*

### Psychological disadvantages

Psychological factors were also described as important reasons for declining to take the depot formulation. Many participants spoke of an unwillingness, anxiety, or fear of switching away from a medication that they perceived to have worked for them. Examples included: *“Why change something that is working?”, “Why mess about when it’s working? That’s really stupid”*, and *“I think it’s working well as it is. So why change something that has meant that I, yes, have been drug free up to now?”* Comments of this kind were common among both those who had declined the depot formulation and some who had agreed to take it, but who had then discontinued the treatment.

Many of the participants who declined the treatment described feeling a psychological need to take their medication every day – *“It feels good to take something every day”,* and that it would otherwise feel like *“there was something missing”.* Some described their daily medication routine as a *“morning ritual”* that involved more than merely taking medication – the routine provided a sense of security that gave them a feeling of control. *“It’s, this morning ritual so to speak. [ …] It maybe sounds a bit silly but it, we drug abusers are people of habit, so taking this medication becomes a habit of course.”* Several participants were convinced that the daily routine was an important reason for their having managed to stay away from drug use and were therefore afraid of relapsing if they were to switch to the depot formulation. *“It sits quite deep, that you have to take your medication every day. That I have to do it because it keeps it in check so much. Yes, but even if I don’t feel good on Suboxone, it keeps something in me in check, that ‘okay, but I can, I can manage one more day, I can do that.’ Yes, I mean it’s really strange, but it totally takes over; the psychological takes over completely. [ …] I can’t risk, I mean I risk [losing] my children if I; I can’t take my medication once a week, I have to take my medication every day.”* For some who had been ambivalent towards the depot formulation, the sense of security associated with having a medication that worked and that they took every day had been the decisive factor, despite them seeing benefits associated with the depot formulation. *“The sense of security, like that you take your medication every day. But it’s a psychological thing, knowing that you’ve taken your medication and that things feel good for that reason. But as I said, it’s both good and bad; with the Buvidal it would probably have gone smoothly, yes, [ …] but I don’t know how I would have felt then, with this psychological thing; that, yes, if it had worked well and you were satisfied, so to speak, with only having an injection once a month, then it would probably have been good.”*

There were also participants who expressed skepticism about switching to a medication that they perceived to be untested and perhaps unsafe. The depot formulation was described as new, insufficiently researched, and to have unknown and potentially dangerous side effects. *“No, I just didn’t want to take that injection. It’s still so new; there is so little research on it too. There was a guy who went there. He came in one day; he had red rings all over his stomach and had had an allergic reaction. And I have an awful lot of allergies myself too. So, I’d rather go in two days a week to collect my medication.”* Several participants said that they didn’t want to be *“guinea pigs.”* In contrast to this, methadone and sublingual buprenorphine formulations were described as *“safe bets”,* whose effects and side effects were well known.

### The pharmaceutical atmosphere

Switching medications can create a great deal of frustration and worry among OAT patients, particularly if the patients experience the change as being forced upon them or as not having a choice in the matter. The introduction of buprenorphine-naloxone (Suboxone) as a replacement for mono-buprenorphine led, for example, to conflict, opposition, and the spread of rumors, particularly in contexts where patients were forced to switch medications [27, 32, 33].

In most places from which we recruited interview participants, there was little sign of this type of problem. The dominant view among the participants was that the introduction of the depot formulation had been conducted in a correct and responsible manner. The majority felt that they had received sufficient information from staff before deciding whether they wanted to test the depot formulation. Information had been given both in writing (in the form of a brochure) and verbally, often by their contact persons. Many felt that the staff had encouraged them to try the depot formulation, but that they had also been warned that there might be difficulties with abstinence symptoms during the first weeks. The possibility of having the next dose early if necessary was described as something positive, and most participants appear to have availed themselves of this possibility. However, several of those who declined the treatment said that the information about the depot formulation sounded *“too good to be true”.*

In addition to receiving information from staff, several said that they had *“asked around”* among other patients who had already tried the depot formulation, to obtain information about their experiences. In all locations there were both positive and negative stories circulating, which some participants felt had created stress and difficulties. *“No, but then I got in touch, my ex had Buvidal, and then I have another friend who’s had Buvidal, and one more friend who’s had Buvidal, who haven’t felt that it worked well for them. And then to begin with, I asked around a lot when I came here. And then [the contact person] told me to ‘stop asking around because it’s so … , first of all I’ve met people who it doesn’t work for, but for the majority it works, and then it’s so individual, so stop asking others and try to have your own experience instead.’ So, then I stopped asking around.”*

There were also cases of negative rumors being spread about the depot formulation, but in most places, there appeared to be a trust between patients and staff which limited the spread of these rumors. There was however one clear exception to the predominantly positive picture described by the interview participants. This related to a clinic where there was substantial dissatisfaction among the participants and a clear distrust in relation to the staff. Participants from this clinic stated that they did not trust the staff and that they couldn’t be honest about problems they were experiencing. Several expressed a fear of being punished by getting their dose reduced, their medication withdrawn, or ultimately by being discharged from treatment. Most participants from this clinic stated that they had initially been offered the possibility of trying the depot formulation, but that those who had declined had then been forced to switch to the new medication. *“I spoke to my contact the day before yesterday, and I said, ‘It gets very stressful and confusing; it’s different messages the whole time.’ Then I think, the same as with the Buvidal, when I was going to start with that it was a choice; you know, you got to choose whether you wanted to. But then in the end, they come along one day and just, ‘No, you have to switch to the injection now,’ like, ‘Ah, okay then.’”*

The participants from this clinic said that they did not trust the staff and that this had a major effect on how they experienced the introduction of the depot formulation. Several described being told that they could have a new dose when they needed it, if the depot formulation did not last the entire week, but that this offer had then shown itself only to be available for a limited period, during the very first weeks. Several participants from this clinic also spoke of having tried for a long time to switch back to their former medication, but in vain. Several participants said that switching back was only possible in the case of a ‘major relapse’, and that this had caused problems. *“Many think, like me, that the Buvidal was crap. You know, many fell back into drug abuse. I mean we’ve never had such a high, how can I put it, a high proportion who are using on the side as there are now. And of course, that’s because we’ve been forced to take that injection.”*

There was also an extensive circulation of negative rumors about the depot formulation at this clinic. Among other things there were rumors about serious side effects that the staff were said to have suppressed. The introduction of the depot formulation at this clinic had led to what Bartoszko [27] has referred to as a pollution of the pharmaceutical atmosphere. The trust that several patients had previously felt in relation to staff had been replaced by distrust. We return to this in the Discussion section.

An overview of the themes and sub-themes identified in the analysis is provided in Table 1 below.

**Table 1:** Themes and sub-themes identified in the analysis

Theme	Sub-theme	Mentioned by
The effects and side effects of the depot-formulation	Withdrawal symptoms during induction period	Most patients
	Other side effects during induction period	Some patients
	Lasting withdrawal symptoms and drug cravings	Patients who had discontinued depot injections
	Lumps at injection sites	Many patients, but mentioned as big problem by some who had discontinued depot injections
Social and practical factors	Freedom of not having to attend the clinic as frequently	Many patients, patients with employment
	More time for family and leisure activities	Many patients
	Benefit not having to handle medication	Patient who accepted depot injections
	No social pressure to divert	Patient who accepted depot injections
	Frequent clinic visits a routine that gives structure to everyday life	Patients who declined depot injections
	Frequent clinic visits a valued social activity	Some patients
	No practical benefits of depot injections	Some patients who collected their medication at pharmacies
Psychological benefits	A shift in self-perception – feeling more “free” and “normal”	Patient who accepted depot injections
	A relief not having to think about the medication every day	Patient who accepted depot injections
	Reduced drug stigma	Patient who accepted depot injections
	The clinic as a risk environment – reduced exposure to patients in active drug use	Patient who accepted depot injections
Psychological disadvantages	Unwillingness and/or fear to change an effective medication	Patients who declined depot injections
	Perceived need to take medication every day	Patients who declined depot injections
	Depot injections perceived as untested with unknown side-effects	Patients who declined depot injections
Pharmaceutical atmosphere	Depot injections were introduced responsibly by staff	Most patients
	Trustful relationships with staff important when deciding on medication	Many patients
	Information and rumors among patients may influence perceptions of depot injections	Many patients
	Coercion leads to dissatisfaction, mistrust and negative rumors	Patients at one clinic

## Discussion

In this study we have explored patients’ experiences with buprenorphine depot injections in seven OAT clinics in Sweden using qualitative interviews. We recruited patients currently receiving depot injections, patients previously treated with depot injections who had stopped, and patients who had been offered depot injections but declined them.

In general, the patients continuing with depot injections expressed high treatment satisfaction. The main benefits they highlighted with the depot injection – social and practical benefits such as increased freedom in everyday life, greater opportunities to decide over their own time, and increased opportunities to travel, as well as psychological benefits such as an increased sense of freedom and “normality”, a reduced stigma, and a shift in self-perception – are well in line with previous research on user perspectives, both among opioid users prior to the launch of depot injections and among patients who have received such treatment [20, 22–24].

Most patients described an initial period of instability, characterized by increased withdrawal, shorter than expected effect span, and side effects. Providing information and support and receiving subsequent injections somewhat earlier during the medication induction period helped participants cope with these initial problems. The time for reaching dose stability roughly corresponded to the time to steady state [34]. Once this was achieved, most patients who continued with depot injections in our sample described increased effect stability compared to sublingual treatments. These patients also reported feelings of support from, and trust in, clinical staff.

Patients who chose to discontinue depot injections often did so early in the treatment process, describing experiences of withdrawal and cravings, as well as side effects similar to those described by patients who had continued depot treatment. An individual variability in how these initial negative aspects of the treatment were perceived may have contributed to the choice to discontinue treatment. The underscores the importance of the clinical use of medication to treat withdrawal with earlier doses, bolus doses or sublingual tablets (as recommended for the respective depot formulation) in these initial stabilizing phases of depot medication.

It also suggests that better information and support, during the initial critical weeks/months, and providing help to distinguish between a lack of effect and side effects may help more patients cope with initial problems until steady state is reached.

The patients who had declined depot injections (and several of those who had felt coerced to start such treatment) cited psychological disadvantages as their main reasons. They felt anxious about making changes to a treatment that they felt was working for them and described daily medication as a positive routine that provided a sense of security. This is in line with previous research on opioid users’ willingness to try depot injections [20, 22], suggesting that depot injections may not be a medication that suits all patients’ needs and expectations.

Some of the reluctance, however, may be explained by depot buprenorphine being new in Sweden when the interviews were conducted, as reflected in the interviews (an “untested medication”, “I don’t want to be a guinea pig”). Many patients had little or no information regarding the depot treatment, and there was uncertainty among patients and peer networks regarding the role of the new medication. Similar issues and concerns were experienced when sublingual buprenorphine and later when buprenorphine-naloxone were introduced, both of which now are trusted medications within peer networks.

Many OAT patients and others with substantial experience of illicit drugs are accustomed to the daily use of various substances to affect their mood and social abilities, to reduce anxiety, improve stamina and so on. Sociologist Philip Lalander [35] coined the term “medical mind” to describe the logic whereby drug users come to consider that many of life’s problems can be solved through the use of medications and/or illicit substances. Having been socialized into a drug-focused culture, they have developed a sophisticated understanding of the functions and uses of different substances, and they have developed specialist skills in using substances to help deal with the various challenges of everyday life. Clinical experience suggest that many OAT patients take this mindset with them when they enter treatment, and it is a mindset that can be very difficult to put aside. Our results show a great variability in participants’ attitudes to their “medical mind”. Whereas some patients described the relief they felt at no longer having to think about their medication every day, others have difficulties envisaging an existence in which “fine-tuning” with the help of their medication is no longer possible. Many patients who declined depot injections described the importance of daily rituals and routines linked to medication, or of the variation in effect during the day. This is in line with earlier qualitative research identifying the breaking of rituals and habits as one of the factors that influences willingness to receive depot formulations [20].

Before making a choice on whether to try depot injections or not, patients sought information from both clinical staff and peers, as suggested by earlier qualitative research [21]. Conflicting information contributed to confusion and uncertainty. Clinical staff awareness of rumors and a willingness to support patients may facilitate decision making.

Interestingly, at one of the sites we identified a distinct negative attitude towards depot injections and a reported lower retention rate. Patients here experienced that staff had not honored initial promises of flexibility regarding when the next doses could be taken or the possibility of switching back to previous medications. Depot injections became mandatory when patients had been promised a choice. This created mistrust between patients and staff, which produced negative behaviors, as indicated by informants reporting that the only way to switch to sublingual medication was through having a “severe relapse”. These findings were in line with the “polluted therapeutic atmosphere”, described by Bartoszko [27] regarding an inadequate introduction of buprenorphine-naloxone. According to the author, forced transfers “fostered a sense of disrespect, failure, and mistrust in patients” (p. 282). Similar phenomena have previously been described by other medical anthropologists exploring placebo-nocebo effects [36].

Our interpretation is that the emergence of what Bartoszko [27] described as a “polluted” pharmaceutical atmosphere at least in part explains the negative attitudes described by participants at one of the sites, with high rates of perceived side effects and conflicts with clinical staff. It is important for clinicians to be aware of this phenomenon. Clinically, this highlights the importance of working with patients’ trust, keeping promises, setting clear boundaries, and using motivational methods and relapse prevention in a mindful way at all levels, to avoid the risk of furthering negative behaviors. Clinicians may also consider actively asking about patients’ knowledge and perceptions of different types of medication, in order to discover the emergence of these types of negative attitudes and narratives at an early stage, and provide alternative, scientifically and clinically accurate information. By inviting patients to explore and relate to their own experiences, and by using a non-confrontative, motivational interview-based approach, clinicians may assist patients in identifying relevant information.

### Limitations

Our results are probably influenced by the treatment setting. Sweden has relatively low access to treatment and a strictly regulated OAT program, with compulsory, daily, supervised intake for at least 3 months, and in practice often much longer in the case of continued substance use. Although access to treatment in Sweden has much improved since revision of the national guidelines in 2015 [11], in line with international consensus [9], access may still be limited by lack of staff. This probably increases the perceived freedom and agency experienced with depot injections and may limit the generalizability of our finding to other settings with more liberal access to treatment. The interview participants were relatively more stable compared to the average for OAT patients in Sweden [31], and the results might therefore be different in a more unstable patient group, with ongoing substance use, and with more pronounced social problems. Depot injections are well established within psychiatry, improving treatment outcomes and reducing the need for hospitalization in schizophrenia, especially in unstable patients with low treatment adherence [37]. Within addiction services, clinical experience with long-acting formulations is limited, but the potential for improving access to OAT is considerable. More research is needed to explore the experiences of patients at the more severe end of the spectrum.

## Conclusions

Buprenorphine depot injections are an important new treatment option in opioid agonist treatment. For many patients, depot injections may have social, practical, and psychological benefits compared to other formulations. However, depot injections are not perceived as an attractive option by all patients. Some are reluctant to try such treatments with reference to psychological factors, while others discontinue treatment due to drug cravings and negative side effects.

Clinical staff appear to play an important role in how patients will perceive their medication. Sufficient information, good relationships with treatment staff, and the possibility of adjusting the dosing schedule when patients experience insufficient effects may contribute to better treatment experiences for patients treated with depot injections. Policy makers need to consider allocating resources to staff education to improve adherence and support during the first period of depot medication. Given the powerful effects of the pharmaceutical atmosphere, being aware of and addressing potential negative aspects that may pollute this atmosphere should be considered when introducing new medications in order to avoid unnecessary treatment discontinuation and improve adherence and treatment results.

## Supplementary Information

**Additional file 1.**

**Additional file 2.**

## Data Availability

The data used in the study are not publicly available due to restrictions made by the Swedish Ethical Review Authority.
