# Supplementary material for: Patient perspectives on depot buprenorphine treatment for opioid addiction – a qualitative interview study
Source: Subst Abuse Treat Prev Policy. 2022 May 25;17:40. doi: 10.1186/s13011-022-00474-2 (PMC9131643; doi:10.1186/s13011-022-00474-2)
Supplement: Supplementary file 1 — Additional file 1. [file 13011_2022_474_MOESM1_ESM.docx]

Supplementary material 1. Interview guide (English translation).

| 1. Background, history of substance use, and related problems 2. Treatment experience, medical and psychosocial, other than opioid agonist treatment (OAT) 3. Experience of and views on OAT treatment 4. Relationship with treatment staff 5. Control vs. support in the treatment relationship 6. Thoughts about choice of medication    1. Positive and negative aspects/ experiences of buprenorphine depot injections    2. Positive and negative aspects/ experiences of sublingual buprenorphine-naloxone combination treatment    3. Positive and negative aspects/ experiences of sublingual mono-buprenorphine treatment    4. Experience of methadone (if any)    5. Psychological aspects of choice of medication    6. Need for support versus control    7. Contact with treatment providers in view of choice of medication    8. Information and experience of control in medication choice    9. Duration of depot injection treatment    10. Effect duration in patients’ own experience.    11. Experience of dosing, effect, cravings during buprenorphine depot injection treatment    12. Routines and habits associated with medication    13. Thoughts regarding control, diversion, misuse (injection, snorting) in relation to medication choice 7. Patients’ perceptions of staff’s views on various medication alternatives 8. Thoughts on the future – continued OAT treatment, possible thoughts about treatment discontinuation |
| --- |

**Supplementary material**

Participants’ characteristics

|  | Total | Ongoing depot treatment | Discontinued depot treatment | Declined depot |
| --- | --- | --- | --- | --- |
| Total participants, N (%) | 32 (100%) | 14 (44%) | 11 (34%) | 7 (22%) |
| Age (years), mean (range), median | 36.6 (21-67), 35 | 32.6 (25-50), 30 | 41.3 (28-67), 36 | 37.3 (21-53), 38 |
| Sex, male (%) | 22 (69%) | 11 (79%) | 7 (64%) | 4 (57%) |
| No. of years in OAT, mean (range), median | 3 (0-20), 2 | 1.9 (0-6), 1 | 2.1 (1-14), 2 | 5.3 (1-20), 2 |
| Opioid addiction duration, mean (range), median | 14.5 (6-29), 15 | 13.0 (6-20), 12.5 | 15.2 (8-25), 15 | 16.4 (6-29), 17 |
| *Current medication* |  |  |  |  |
| Buvidal (weekly) | 5 | 5 | - | - |
| Buvidal (monthly) | 8 | 8 | - | - |
| Sublocade (monthly) | 1 | 1 | - | - |
| Buprenorphine | 12 | - | 9 | 3 |
| Buprenorphine-naloxone | 5 | - | 1 | 4 |
| Methadone | 1 | - | 1 | - |
| *Main substance of use before starting OAT* |  |  |  |  |
| Heroin | 7 | 1 | 3 | 3 |
| Heroin and other opioids | 15 | 10 | 4 | 1 |
| Other opioids | 8 | 2 | 4 | 2 |
| Takes opioids daily but names other substances as main substance of use | 2 | - | 1 | 1 |
| *Treatment experience other than OAT* |  |  |  |  |
| Extensive^1^ | 20 | 10 | 6 | 4 |
| Limited^2^ | 10 | 2 | 6 | 2 |
| None | 2 | 1 | - | 1 |
| *Current substance use* |  |  |  |  |
| Stable remission (no use >12 months) | 12 | 5 | 6 | 1 |
| Recent remission (3-12 months) | 11 | 4 | 2 | 5 |
| Ongoing use | 8 | 4 | 3 | 1 |
| No information on current use | 1 | 1 | - | - |

1. Extensive treatment experience: psychosocial and psychological treatment, abstinence-based inpatient treatments including residential and/ or compulsory treatment
2. Limited treatment experience: tried abstinence-based treatment at least once
