# Supplementary material for: Patient perspectives on depot buprenorphine treatment for opioid addiction – a qualitative interview study
Source: Subst Abuse Treat Prev Policy. 2022 May 25;17:40. doi: 10.1186/s13011-022-00474-2 (PMC9131643; doi:10.1186/s13011-022-00474-2)
Supplement: Supplementary file 2 — Additional file 2. [file 13011_2022_474_MOESM2_ESM.docx]

Supplementary material 2. Table of participants’ characteristics.

|  | Total | Ongoing depot treatment | Discontinued depot treatment | Declined depot |
| --- | --- | --- | --- | --- |
| Total participants, N (%) | 32 (100%) | 14 (44%) | 11 (34%) | 7 (22%) |
| Age (years), mean (range), median | 36.6 (21-67), 35 | 32.6 (25-50), 30 | 41.3 (28-67), 36 | 37.3 (21-53), 38 |
| Sex, male (%) | 22 (69%) | 11 (79%) | 7 (64%) | 4 (57%) |
| No. of years in OAT, mean (range), median | 3 (0-20), 2 | 1.9 (0-6), 1 | 2.1 (1-14), 2 | 5.3 (1-20), 2 |
| Opioid addiction duration, mean (range), median | 14.5 (6-29), 15 | 13.0 (6-20), 12.5 | 15.2 (8-25), 15 | 16.4 (6-29), 17 |
| *Current medication* |  |  |  |  |
| Buvidal (weekly) | 5 | 5 | - | - |
| Buvidal (monthly) | 8 | 8 | - | - |
| Sublocade (monthly) | 1 | 1 | - | - |
| Buprenorphine | 12 | - | 9 | 3 |
| Buprenorphine-naloxone | 5 | - | 1 | 4 |
| Methadone | 1 | - | 1 | - |
| *Main substance of use before starting OAT* |  |  |  |  |
| Heroin | 7 | 1 | 3 | 3 |
| Heroin and other opioids | 15 | 10 | 4 | 1 |
| Other opioids | 8 | 2 | 4 | 2 |
| Takes opioids daily but names other substances as main substance of use | 2 | - | 1 | 1 |
| *Treatment experience other than OAT* |  |  |  |  |
| Extensive^1^ | 20 | 10 | 6 | 4 |
| Limited^2^ | 10 | 2 | 6 | 2 |
| None | 2 | 1 | - | 1 |
| *Current substance use* |  |  |  |  |
| Stable remission (no use >12 months) | 12 | 5 | 6 | 1 |
| Recent remission (3-12 months) | 11 | 4 | 2 | 5 |
| Ongoing use | 8 | 4 | 3 | 1 |
| No information on current use | 1 | 1 | - | - |

1. Extensive treatment experience: psychosocial and psychological treatment, abstinence-based inpatient treatments including residential and/ or compulsory treatment
2. Limited treatment experience: tried abstinence-based treatment at least once
